# Challenges for health systems seeking to embrace virtual health care for population health

**DOI:** 10.1007/s10198-022-01503-4

**Published:** 2022-08-03

**Authors:** Panos Kanavos, Michelle Vogelsang, Madeleine Haig, Vasiliki Kolovou

**Affiliations:** 1 Department of Health Policy and LSE Health - Medical Technology Research Group (MTRG), London School of Economics, Houghton Street, London, WC2A 2AE UK; 2 Department of Health Policy and LSE Health - Medical Technology Research Group (MTRG), London School of Economics, London, UK; 3 King’s College NHS Foundation Trust, London, UK

**Keywords:** Digital health, Virtual health care, Population health, Health care reform

## Abstract

A gradual move to proactive illness prevention requires a strategic shift towards population health management by health care systems. Such a shift becomes necessary to improve outcomes, reduce inequalities and manage costs better, as life expectancy increases and chronic illness becomes more prevalent. Health system digitisation and greater focus on virtual health care (VHC) can contribute to active population health management. For that to happen, health systems need to address and overcome several challenges currently preventing the rapid introduction and scale up of VHC for population health; these include implementing changes in care models and focus on digitally enabled population health approaches; addressing culture and mindset barriers; resolving regulatory bottlenecks; overcoming technical limitations, inter-operability and data security issues; and, finally, aligning stakeholder incentives and expectations.

## Background

A move from reactive to proactive illness prevention through population health management is the future of healthcare systems as life expectancy increases and chronic conditions become more frequent. Pre-existing weaknesses and gaps in many healthcare systems, including shortages in clinical supplies and personnel, under-investment in public health infrastructure, and a lack of coordination and agility amongst policy-makers and political authorities may reduce the impact population-level health interventions could have. Virtual healthcare (VHC) has a space in helping care providers to efficiently and cost-effectively target the right patients, facilitate care coordination and patient communication, and support and empower patients to manage their care and prevent future disease.

Whilst there is no single accepted definition of population health (PH), it is defined as health outcomes of a group of individuals, including the distribution of such outcomes within the group, as well as a methodology for identifying those at risk of ill health (both physical and mental) and the application of the appropriate interventions for prevention or care and rehabilitation. It balances the intensive management of those in the greatest need of health care with preventative and personal health management for those at lower risk levels. Population health management (PHM) works towards improving PH by data-driven planning and delivery of proactive care to achieve maximum impact via segmentation, stratification and impact modelling to identify local ‘at risk’ cohorts. In principle, the resulting targeted interventions prevent ill-health, improve care and support for people with ongoing health conditions and reduce unwarranted variations in outcomes. The key components of a successful population health programme can be summed up as data aggregation, patient stratification, care coordination, patient engagement, performance reporting, and good administration.

Clearly, digital tools have a key role to play in expanding population health. Virtual health encompasses several modalities of digital and telecommunication technologies that may be used to deliver healthcare, help enhance access, improve value, personalise care and establish a competitive advantage by operationalizing technology solutions. Specifically, virtual health has the potential to reduce gaps in care by allowing patients to access physicians remotely without having to rely on scheduled, routine visits, helping patients to seek care when most needed and reducing the number of ‘high cost’ visits or emergency hospital admissions. It also has the capacity to bridge the gap between specialists by coordinating patient needs with multiple specialists and enhancing integrated care as well as allowing those in remote areas to effectively access healthcare [1]. Some now view it as a care delivery transformer [2].

## Key challenges to leveraging virtual health care for population health

Despite the likely benefits of such a paradigm shift, there are several challenges preventing the rapid introduction and scale up of VHC for population health. Key among them include, changing care models and focussing on digitally enabled population health approaches; eliminating culture and mindset barriers; ironing out regulatory bottlenecks; addressing technical limitations, inter-operability and data security; and, rectifying misaligned stakeholder incentives. We explore these, in turn.

### Changing care models and focussing on digitally enabled population health approaches

Traditional care models prioritise acute care and health care providers are generally seen as responsible for an individual’s health. This is particularly true for publicly funded health systems which may be described as “paternalistic” where patients (taxpayers) may feel less responsible for their own health. This could reduce the effectiveness of a population health approach that relies on creating informed and empowered patients to facilitate self-care and reduced reliance on advanced clinical interventions.

Population health management constitutes a fundamental shift in how healthcare is traditionally managed. A shift to a digitally enabled population health approach may be challenged by the health system’s existing structure in terms of administrative and financial boundaries. Even centralised health systems are usually set up with administrative boundaries and existing fragmentations could impact the effectiveness of population-based care. For example, in England, a move to population health management required establishing new Integrated Care Systems (ICS) [3, 13]. Previously existing health and social care services were provided by separate bodies—Local Authorities (who provide social care) and CCGs (who focus on health care). These bodies did not always share the same administrative boundaries, lacked linked financial incentives such as similar budget constraints, and were limited in how they could jointly commission or plan services [4]. By establishing ICS, England’s Department of Health and Social Care hopes to better coordinate care within regions.

### Eliminating culture and mindset barriers

On a basic level, a successful PHM programme requires clinicians and, more generally, healthcare systems, to address both acute and chronic conditions. However, healthcare systems and care providers generally manage disease reactively, rather than proactively and lack an embedded focus on the prevention of chronic disease unless appropriately incentivised. As prevention has not historically been prioritised equally with treatment, there needs to be a shift in thinking, organisation, resource use, reporting, and monitoring in health and care delivery. This can be a challenge for some organisations who have been driven by organisational and patient outcome targets that do not strongly focus on prevention efforts [5].

A related challenge is that current health providers are primarily patient-focussed organisations, largely leaving population well-being and public health in the domain of other organisations. This shift in focus to the population-level requires system-wide changes in priorities, work cultures, and physician mindsets around a previously episodic patient relationship.

When population health is coupled with a move towards digitally enabled care there needs to be a cultural shift in beliefs around how quality care is planned and provided. For a health system to embrace virtually supported PHM, providers and patients need to be open to operating digitally. The Covid-19 crisis has facilitated an openness to VHC and digital tools are now more widely accepted; however, in many countries there remains a digital skills gap among service providers and a ‘digital divide’ among patients that could limit the extent of virtual population-based care.

A final cultural shift that could signal readiness to engage in virtually enabled population health management would be the willingness of healthcare systems to engage with stakeholders outside of their structure, such as by exploring public–private partnerships or private provision of digital components, skills, or training.

### Ironing out regulatory and political bottlenecks

Whilst effective regulation is important for patient and provider confidence, it can also frustrate suppliers and patients if it does not move fast enough. Outdated regulatory procedures not equipped to respond to rapid technological advancement may unwittingly hold back potential advancements in clinical treatment, artificial intelligence, and digital health that could improve health outcomes and reduce disparities.

Traditional market entry and take up pathways were designed for pharmaceuticals and medical devices. In some countries, such as Germany, the USA, and the UK, the review procedures for digitally enabled devices are slowly being updated to cater to new technologies; however, there remains uncertainty around the regulatory pathways for digital device approvals particularly with regards to proving clinical and economic benefit [6].

Additionally, current regulatory and value-assessment systems usually focus on products that address existing health conditions rather than prevention. Proving clinical and economic benefit is especially difficult for prevention-focussed digital products which are important tools for population health. Thus, it is essential that further progress is made to establish clearer pathways for regulatory approvals for digital prevention products if prevention is to be prioritised under population health management [7]. Along with challenges in product approval processes, many countries lack a clear and practical coverage or reimbursement strategy for digital tools. Overcoming these challenges could offer a strong signal that healthcare systems are ready to engage on virtual care for PHM.

### Addressing technical challenges, inter-operability and data security

Digital tools and big data can support both the technological and cultural shift towards population health. However, many countries struggle with technical barriers, such as a lack of technical knowledge and access to high level capability, data security and privacy issues, and face other basic technical issues (i.e., low bandwidth) that can affect the adoption of VHC. Expanding focus beyond the care and treatment of specific patients with known problems to identify all the individuals in a population with potential conditions and continually monitoring the status of those at-risk patients takes considerable computing power. Such data access and analyses has historically been difficult due to a lack of robust holistic and longitudinal patient data and the ability to meaningfully process large datasets.

Covid-19 has prompted a positive shift towards increased digital investment and digital skill-building that has spurred the adoption and willingness to use digital tools. Since 2020, both patients/end users and health systems have increasingly utilised VHC where previously there was more of a general reluctance to embrace modern technology and a lack of patient and provider trust in VHC offerings. As virtual health care has expanded, an ongoing criticism is the lack of inter-operability and data security. System inter-operability across different care providers is key for better integrated care and for tracking people within large populations. However, with access to more data and increased use of technology, health systems expose themselves to cyber attacks and data breaches. There are many examples of such attacks in recent history across Europe and the USA. In an effort to address these challenges, policy-makers and regulators have established information, privacy, and security standards; however, such standards do not automatically guarantee tools are interoperable and secure.

### Aligning incentives among stakeholders

Leveraging VHC to contribute to population health management requires aligning incentives across various stakeholders including regulators, policy-makers, service purchasers, healthcare providers, and patients or carers. These stakeholders are understandably driven by different motivations and values which are broadly summarised in Table 1.

**Table 1:** Stakeholder value drivers for the uptake of VHC for population health management

Stakeholder	Primary value driver(s)	Secondary value drivers
Regulators	Safety; Effectiveness	Privacy; Security; Inter-operability; Other minimum operating requirements
Policy-makers	Quality of care; Financial stability	Health inequality reduction
Purchasers	Cost-effectiveness; Improved member experience; Effectiveness; Budget impact	Big data; Long-run cost management (macro-economic efficiency)
Healthcare Providers	Care quality; Patient relationships; Reimbursement	Targeted health care delivery; Chronic disease prevention; Self-management
Patients/Carers	User experience; Perceived health benefits	Credibility; Convenience

Source: The authors

Regulators’ priorities are understood through the virtual health product assessment criteria from agencies in the USA, UK, and Germany set the minimum expected parameters for digital tools and establish the importance of privacy, security, inter-operability, and clinical benefit. However, underlying these minimum standards, regulators are motivated to support the introduction of innovative products that are safe and effective [8]. Those responsible for setting policy and health strategy rely on the oversight provided by regulators and are motivated by a desire to provide quality care, ensure financial sustainability, and, in some environments, to meet people’s health needs and reduce health inequalities [3, 5]. On the other hand, health purchasers are primarily motivated by cost-effectiveness, budget impact and patient experience and see potential in population-based VHC to improve on these [9]. Data on patient populations are also highly valued by purchasers and commissioners of care as they have the potential to provide insights to funders, allowing them to take a data-driven approach to premiums, resource allocation, and co-payments. Effective population-based interventions that prevent chronic disease and support self-management are also valued as these can help manage long-run costs [10].

The final stakeholders to include in population health shifts are providers and patients. When producing and marketing a VHC product, suppliers often need to consider two customer types: providers, who often act as gatekeepers for products, and patients or carers, who may interact with and use the product. Healthcare providers generally place trust in regulatory bodies to ensure that available products meet safety and effectiveness requirements. Where these are certain, providers are concerned about ensuring patients’ privacy and respect, building trust with patients, and delivering the same or improved standard of care as face-to-face service without placing additional demands on their time [10]. For patients, user experience was identified as one of the main success factors for patient-facing platforms, followed closely by credibility/trust and perceived health benefits [11].

## The road ahead

As we move further into the twenty-first century the healthcare landscape is primed for the expanded adoption of virtual healthcare, particularly within the remit of PHM. By 2025, global spend on digital health is predicted to reach €1 trillion and digital products and services will grow to a market share of 12% [12], leaving little doubt that virtual health and digital platforms will transform healthcare organisation and delivery in the coming years. Decision-makers are eager to establish financially sustainable systems that stem the rise of costly, preventable disease. If the five sets of challenges highlighted here are addressed, health systems may be well on the way to expanding VHC for population health and improving health outcomes.
